# Cambial variations of *Piper* (Piperaceae) in Taiwan

**DOI:** 10.1186/s40529-017-0172-z

**Published:** 2017-03-29

**Authors:** Sheng-Zehn Yang, Po-Hao Chen

**Affiliations:** National Pingtung University of Science and Technology, No. 1, Shuefu Rd., Neipu, Pingtung, 912 Taiwan, ROC

**Keywords:** Cortical vascular bundle, Liana, Mucilage canal, Piperaceae, Stem anatomy

## Abstract

**Background:**

Cambial variations in lianas of Piperaceae in Taiwan have not been studied previously. The stem anatomy of seven *Piper* species from Taiwan was examined to document cambial variations and better distinguish the species when leaves are absent.

**Results:**

A key for the seven species is provided, based on the internal stem anatomy. The seven *Piper* species climb via adventitious roots, and in cross section, the stems were generally eccentric and oblate, although a transversely elliptic stem was found in *P. kadsura* (Choisy) Ohwi and *P. sintenense* Hatus. A cambial variant with secondary growth of external primary vascular bundles and xylem in plates was observed in all species except *Piper betle* L., which developed another cambium variant with xylem furrowed deeply by parenchyma proliferation. The sclerenchymatous ring surrounding the medullary vascular bundles was always continuous except in *P. betle,* where it was discontinuous. Mucilage canals varied from absent to present in the center of the pith, or present in the pith and inner cortex. Different sizes of vessels dispersed throughout the stem were ring or diffuse porous. The numbers of medullary and peripheral vascular bundles were distinctive and the widths of rays were noticeably different in each species. Differences in the growth rate of the medullary vascular bundles produced two development types of vascular bundles, although in both types, the peripheral vascular bundles gradually lengthen and become separated from each other by wide rays.

**Conclusions:**

We documented the internal stem anatomy of six previously unstudied species of *Piper*, including three endemic species, *P. kwashoense* Hayata, *P. sintenense*, and *P. taiwanense* Lin and Lu, and found that *P. betle* had deeply furrowed xylem, which had not been reported for the species before. The descriptions and photographs of seven *Piper* species will also provide a basis for further morphological studies.

**Electronic supplementary material:**

The online version of this article (doi:10.1186/s40529-017-0172-z) contains supplementary material, which is available to authorized users.

## Background

Lianas usually have two stages of development, the autologous support stage and the climbing stage (Schnitzer and Bongers 2002). Internally, the autologous support stage is characterized by a few narrow vessels and many thick fibers. The climbing stage involves secondary growth of the stem, which can be a standard or anomalous (Isnard and Silk 2009). Morphological variation in liana stems is primarily associated with the geometry of the phloem and xylem, and irregular shapes within the stems are classified into cambial variants (Angyalossy et al. 2015). The diversity of liana stem shapes, structures, and cambial variants has been recently reviewed (Angyalossy et al. 2012), including those of Piperaceae.

Members of Piperaceae represent about five genera and 3700 species around the world (Christenhusz and Byng 2016), and many are economically important because of their medicinal and culinary uses. They are erect or scandent shrubs, small trees, or succulent, terrestrial herbs, with nodose stems. The leaves are entire, alternate to opposite or verticillate, petiolate or infrequently subsessile, palmately nerved or penninerved, pellucid dotted, and sometime aromatic (Souza et al. 2004). The genus *Piper* L. is represented by about 1050 species distributed primarily in the tropics (Mabberley 2008). The most outstanding anatomical character in the Piperaceae is the nature of the vascular bundles in the stem. In all species of Piperaceae studied to date in the genera *Piper*, *Manekia* Trel., and *Zippelia* Blume, vascular bundles are organized in two or more concentric rings, a characteristic that is not present in other genera in Piperaceae (Trueba et al. 2015).

Several studies have characterized cambial variants in certain *Piper* species. *Piper obtusilimbum* C. DC. has a typical arrangement of tissues (Tepe et al. 2007), with a parenchymatous pith, medullary vascular bundles, a sclerenchymatous cylinder, peripheral vascular bundles, and a vascular cambium. The stem of *Piper betle* L. had an inner irregular circle of primary vascular bundles interspersed with a large mucilage canal in the pith, more mucilage canals in the inner cortex, an undulating wall of sclerenchyma, and an outer ring of smaller cortical bundles. The primary vascular (medullary) bundles occupied the pith (Beck 2011). Raman et al. (2012) showed that the stem of *P. betle* has a ring of mucilage canals located between cortical and medullary rings of primary vascular bundles, a central mucilage canal in the pith, secretory cells in the cortex, cortical fibers, and an endodermis with a Casparian strip. The descriptions of *P. betle* here do not match each other owing to the age of the stem. Beck (2011) showed that the stem of *P. excelsum* G. Forst. had two medullary bundles that occupied the very center of the pith. The species had two cylinders of vascular bundles. The central, irregularly grouped primary vascular bundles were enclosed by a thick cylinder of secondary xylem capped by phloem that is separated by wider medullary rays of primary parenchyma.

Raman et al. (2012) studied *P. sarmentosum* Roxb. The stem of *Piper sarmentosum* has a large central mucilage canal, medullary vascular bundles surrounded by parenchyma in the pith, a ring of cortical vascular bundles, and a discontinuous ring of collenchyma in the outer cortex. Saraswathy et al. (2013) indicated that the stem of *P. retrofractum* Vahl. was circular in cross section, with a large central mucilage canal surrounded by individual bundles scattered in a parenchymatous cortex, which was encircled by a wavy ring of sclerenchyma followed by a ring of vascular bundles and medullary rays. A pericycle and collenchyma formed the outer cortex. In a broader study of woodiness in Piperales and Trueba et al. (2015) presented images of transverse sections of *P. gorgonillense* Trel. and Yunck. and *P. nudibracteatum* C. DC. *Piper gorgonillense* produced wide, lignified rays, with solitary or clustered vessels, but the center and edge of the stem were not included. A growth ring was visible in the rays and vascular tissue. The peripheral vascular bundles in *Piper nudibracteatum* gradually elongated through secondary growth, exceeding the medullary vascular bundles, while the growth of the medullary bundles was minimal, and wide rays were formed by primary parenchyma cells. Santos et al. (2015) showed that in *P. amalago* L., a large parenchymatous pith is surrounded by an inner circle of approximately nine medullary bundles, a ring of sclerenchymatous fibers, and an outer circle of approximately 30 vascular bundles, some of which have bundles of fiber capping the adjoining phloem.

According to these studies and others, *Piper* typically has an inner series of vascular bundles separated from an outer series by a sclerenchymatous ring. This pattern has been observed in *P. nigrum* L. and *P. colubrinum* Link (Ravindran and Remarshree 1998); *P. diospyrifolium* Kunth (Souza et al. 2004); *P. amalago*, *P. betle*, *Piper excelsum*, *Piper gorgonillense, P. nudibracteatum, Piper obtusilimbum*, *P. retrofractum, P*. *sarmentosum*, *P*. *regnelli* (Miq.) C. DC. (Pessini et al. 2003); *P*. *gaudichaudianum* Kunth (Albiero et al. 2005b); *P*. *crassinervium* Kunth (Albiero et al. 2005a); *P*. *hispidum* Sw. (Albiero et al. 2006); *P*. *arboretum* (Souza et al. 2009); and *P. mikanianum* (Kunth) Steud. (Duarte and Siebenrock 2010).

Taiwan is located in the subtropical zone, with a warm and humid climate. Piperaceae are represented in Taiwan by two genera, *Peperomia* Ruiz and Pavon and *Piper*. Eight scandent species and two herbs are recorded in the genus *Piper* (Lin and Lu 1996; Hsu and Chung 2016). However, data regarding the internal stem anatomy of seven of the climbing species are lacking. Cambial variations are diverse within the family, so the present study attempts to (1) provide detailed photographs of features discussed, (2) specify the developmental stages observed, and (3) provide a key based on anatomical characters of transverse sections to facilitate the identification of irregular cambial activity in lianescent species of the genus *Piper* in Taiwan.

## Methods

From 2014 to 2016, we collected multiple samples of seven *Piper* species, which are extensively distributed in the low mountainous forests of Taiwan. Species, collecting localities, voucher numbers, and the maximum and minimum stem diameters are presented in Table 1. Stems with a lignified epidermis were selected to compare and identify the structural variations using the obvious secondary growth characteristics in their cross sections. For each species only one sample, with obvious cambial variations that were easy to observe, was selected for photos and description. The species *P. interruptum* Opiz var. *multinervum* C. DC. was not included in this present study because the collected stem was too small and without obvious cambial structure. The developmental stages of vascular bundles in *P. betle* and *Piper kadsura* were observed with particular care because the fleshy materials we collected included different stem sizes for these two species.

**Table 1:** *Piper* species examined for this study

Species	Collection localities	Herbarium and voucher numbers	Minimum diameter (mm)	Maximum diameter (mm)
*Piper arborescens* Roxb.	Lanyu, Taitung County	PPI77234	3.9	5.5
*Piper betle* L.	Lilongshan, Taiwu, Pingtung County	PPI76665	3.5	32.0
*Piper kadsura* (Choisy) Ohwi	Liangshan, Machia, Pauli, Pingtung County	PPI61858	2.5	15.4
*Piper kawakamii* Hayata	Hsiaoliouchiou, Pingtung County	PPI63612	7.8	7.8
*Piper kwashoense* Hayata^a^	Lanyu, Taitung County	PPI19807	6.1	9.8
*Piper sintenense* Hatusima^a^	Tajen, Taitung County	PPI60014	2.1	4.8
*Piper taiwanense* Lin and Lu^a^	Liangshan, Machia, Pauli, Pingtung County	PPI79479	8.5	15.4

^a^Endemic to Taiwan and distributed at low altitudes

Fresh stems were cut into pieces about 5 cm long, and a freehand cross section of each stem was made with a razor blade. The stem surface was immediately photographed using a Nikon D7100 SLR digital camera (Lens AF Micro Nikon 60 mm 1:2.8D, Nikon Corporation, Tokyo, Japan), and qualitative and quantitative anatomical traits were determined using Image-J software (Ferreira and Rasband 2011). The specimens were dried in an oven (60 °C) for 4–5 days and were then stored at −40 °C for 3–4 days. All specimens were deposited in the Provincial Pingtung Institute (PPI) herbarium at the National Pingtung University of Science and Technology, Pingtung, Taiwan, for subsequent identification. The nomenclature follows Flora of Taiwan Volume 2 (Lin and Lu 1996).

The stem anatomy of *P. betle* and *Piper kwashoense* has been reported in earlier studies (Raman et al. 2012; Yang and Chen 2015), but all photographs and observations presented in this study are new. The climbing mechanisms of the lianas (adhesive, hook, or twining) follow Chen et al. (2013). Terminology for the description follows Chiang (Chiang 1973), Metcalfe and Chalk (1979, 1985), Tepe et al. (2007), Beck (2011), Raman et al. (2012), Saraswathy et al. (2013), and Santos et al. (2015) (see Additional file 1: Appendix), and the stem shape determined by a length/width ratio of between 1.5 and 2.0 followed the Systematic Association Committee for Descriptive Terminology (1962).

## Results

**Fig. 1:**
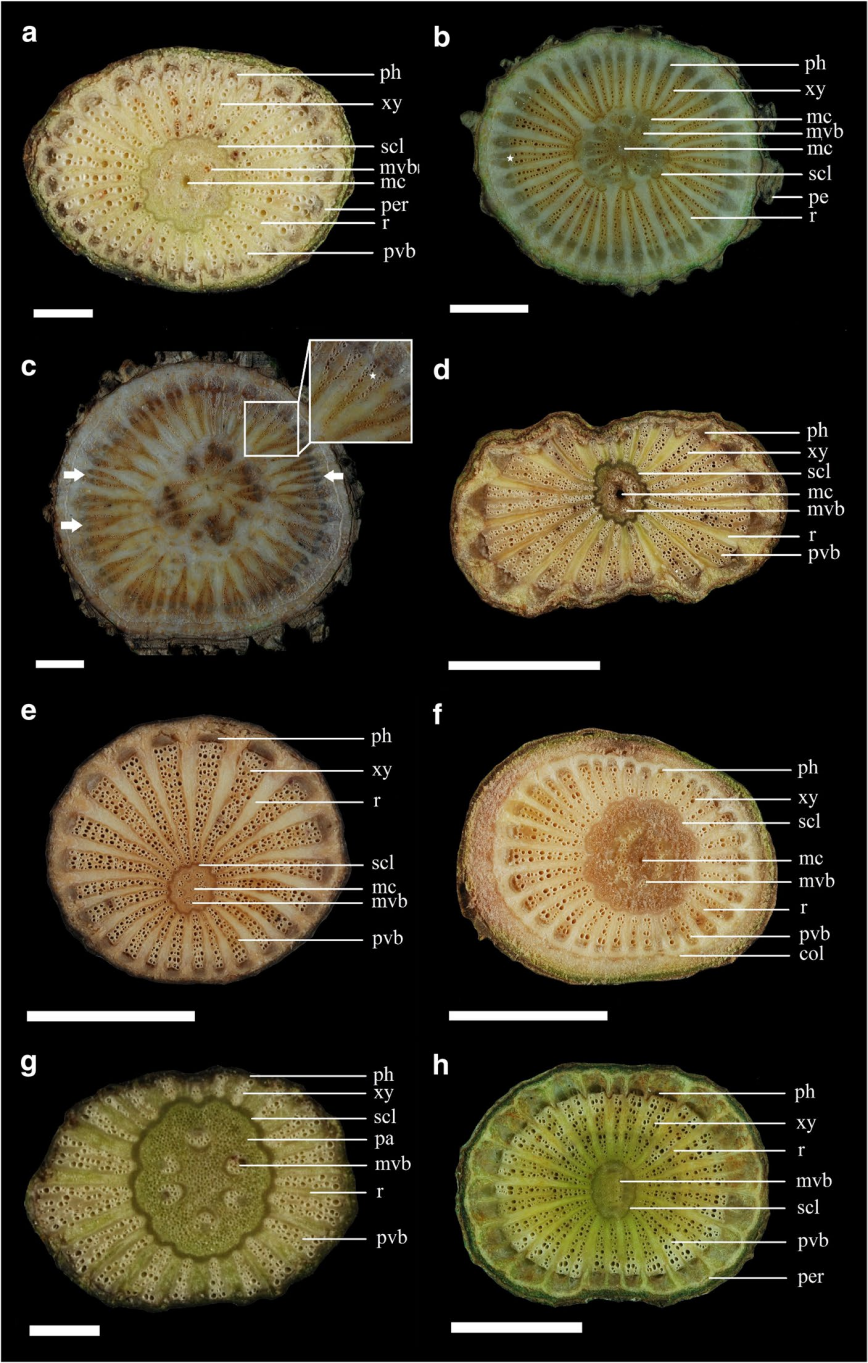
Stem transverse sections of seven *Piper* species. **a** *P. arborescens*, showing only one mucilage canal (mc) in the center of the pith, surrounded by nine medullary vascular bundles (mvb), a continuous sclerenchyma (scl) ring separating the two cycles of vascular bundles, and wide rays (r) separating the peripheral vascular bundles (pvb); approximately 30 pvb; pericycle (per) surrounding the phloem (ph). **b**, **c** *P. betle*. **b** Showing discontinuous scl ring, secondary growth of the mvb and pvb approximately 17 mvb and 37 pvb; periderm (pe) thick and grooved. **c** Deeply furrowed xylem (*arrowhead*) by secondary parenchyma proliferation (*white star*); approximately 13 mvb and 31 pvb, and rays become wider, separating the pvb. **d** *P. kadsura*, showing triangular phloem (ph), and rays (r) which composed of secondary parenchyma; approximately 8 mvb, and 21 pvb. **e** *P. kawakamii*, showing approximately 7 mvb, and 22 pvb separated by wide rays (r). **f** *P. kwashoense*, approximately 12 mvb and 34 pvb, and collenchyma (col) ring surrounding the peripheral regions. **g** *P. sintenense*, showing parenchyma (pa) occupying much of the pith volume, approximately 6 mvb and 22 pvb. **h** *P. taiwanense*, showing approximately 8–9 mvb, 21–26 pvb, and pericycle (per) surrounded the phloem (ph). *Scale*: *Scale* **a** = 1 mm; **b**–**d** = 5 mm; **e**, **f**, **h** = 5 mm; **g** = 1 mm

**Fig. 2:**
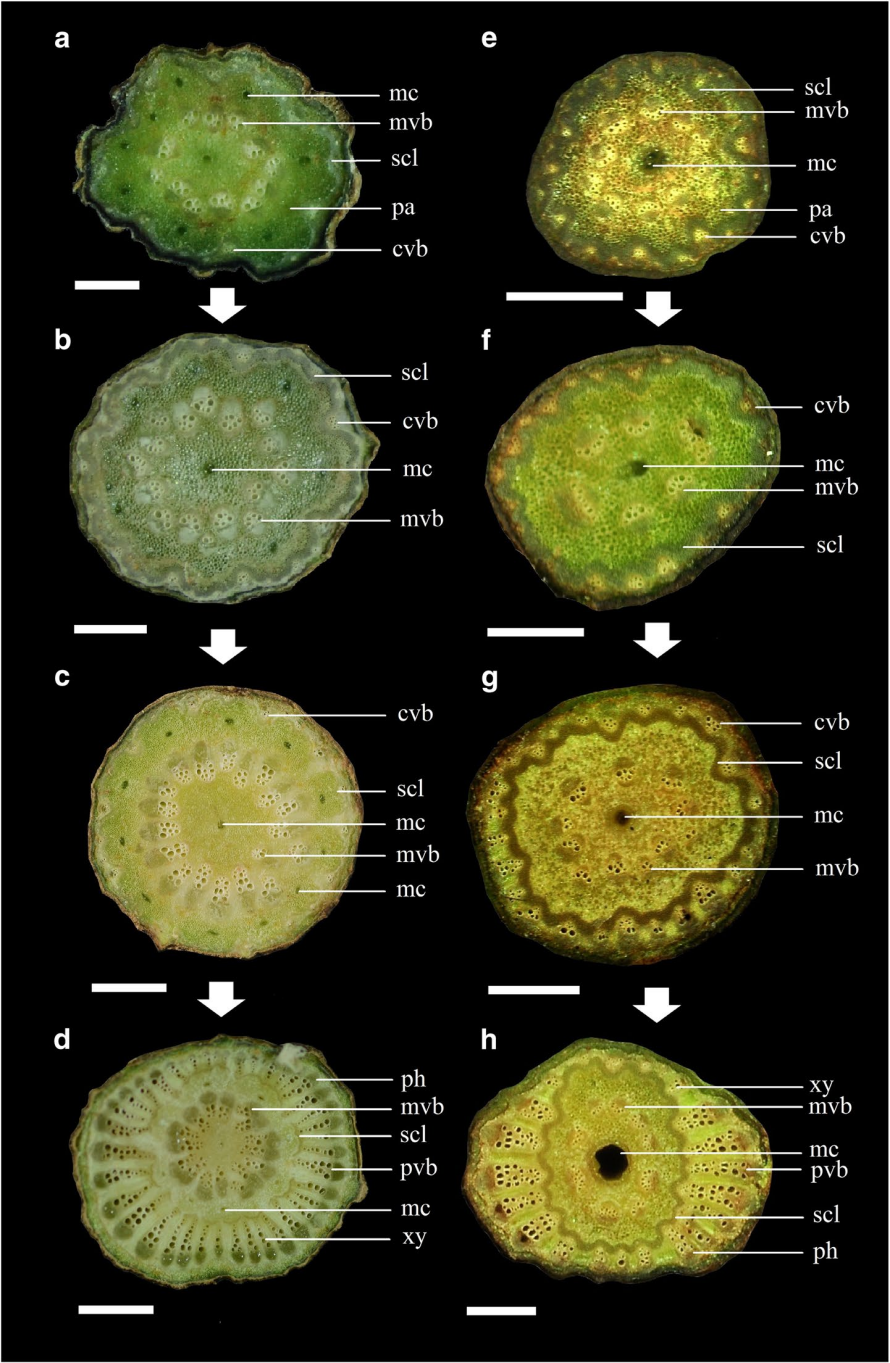
Two development types in *Piper* in Taiwan. **a**–**d** Stem transverse sections of development stages in *P. betle*. **a** The pith surrounded by approximately 12 medullary vascular bundles (mvb), a ring of sclerenchyma (scl), and an indistinct number of cortical vascular bundles (cvb) in the outer cortex. **b** The mvb increase in number, approximately 19 mvb and an indistinct number of cvb. **c** The ring of scl begins to separate as the wide rays of secondary parenchyma are initiated, an indistinct number of cvb in the outer cortex. **d** Approximately 18 mvb and 38 peripheral vascular bundles (pvb), mc in the outer cortex, and showing rounded-elongate phloem (ph) and vessels of xylem (xy). **e**–**h** Stem transverse sections of development stages in *P. kadsura*. **e** Approximately 10 mvb and a ring of scl, and the cvb develop apparently. **f** Both mvb and cvb continue to grow, approximately 7 mvb, 20 cvb. **g** The scl ring appears apparently, and secondary production is limited to the cvb, approximately 11 mvb and 24 cvb. **h** Approximately 9 mvb and 22 pvb, all the pvb continue to grow, and become longer than the mvb in length, and showing triangular phloem (ph) and vessels of xylem (xy). *Scale* **a**, **b**, **e** to **h** = 1 mm; **c**, **d** = 2 mm

The anatomical characteristics of the stems are described in Table 2 and shown in the figures as follows: *Piper arborescens* (Fig. 1a), *P. betle* (Figs. 1b, c, 2a–d), *P. kadsura* (Figs. 1d, 2e–h), *Piper kawakamii* (Fig. 1e), *P. kwashoense* (Fig. 1f), *Piper sintenense* (Fig. 1g), and *Piper taiwanense* (Fig. 1h). The stem shape was generally eccentric to oblate or transversely elliptic in cross section due to differential amounts of xylem deposition (following Carlquist 1991). The number of medullary vascular bundles or peripheral vascular bundles varied among species, from approximately 4–7 medullary vascular bundles in *P. sintenense* (Fig. 1g) to 12–19 in *P. betle* (Fig. 1b, c) and approximately 15–23 peripheral vascular bundles in *P. kadsura* (Fig. 1d) and *P. sintenense* (Fig. 1g) to 34–41 in *P. kwashoense* (Fig. 1f). Vessels of different sizes are dispersed throughout the stem and there is no obvious arrangement of vessels in the secondary xylem, so we suggest that they can be classified as ring porous and diffuse porous following Beck (2011).

**Table 2:** Anatomical characteristics of *Piper* stems in Taiwan (transverse sections)

Characteristic	*Piper arborescens*	*P. betle*	*P. kadsura*	*P. kawakamii*	*P. kwashoense*	*P. sintenense*	*P. taiwanense*
Shape	Oblate	Oblate	Transversely elliptica	Oblate	Oblate	Transversely elliptica	Oblate
Phloem bundles	Arcuate	Rounded-elongate	Triangular	Arcuate	Arcuate	Arcuate	Rectangular to triangular
Mvb number	9–13	12–19	5–11	6–7	12–14	4–7	6–9
Pvb number	30–31	30–40	15–23	21–22	34–41	16–23	21–26
Ray width (μm)	37–251	129–617	55–493	38–603	80–294	106–276	66–342
Vessel diameter (μm)	4–128	18–217	9–125	10–167	10–145	7–100	6–197
Vessel arrangement	2	2	2	1	1	2	1
Sclerenchyma ring	1	0	1	1	1	1	1
Furrowed xylem	0	1	0	0	0	0	0
Parenchyma proliferation	0	1	1	0	0	1	1
Mc	1	1	1	1	1	0	0
Mc dispersed in cortex	0	1	0	0	0	0	0
Central mc in pith	1	1	1	1	1	0	0
Pericycle	1	0	0	0	1	0	1

*mvb* medullary vascular bundles, *pvb* peripheral vascular bundles, vessel arrangement: *1* ring-porous secondary xylem, *2* diffuse-porous secondary xylem; sclerenchyma ring, *0* discontinuous, *1* continuous; furrowed xylem: *0* absent, *1* present; parenchyma proliferation, *0* absent, *1* present; *mc* mucilage canals, *0* absent, *1* present; mc in cortex: *0* absent, *1* present; central mc: *0* absent, *1* present; pericycle: *0* absent, *1* present

The vessel arrangement in the secondary xylem was diffuse porous in *P. arborescens* (Fig. 1a), and ring porous in *P. kawakamii* (Fig. 1e). A ring of sclerenchyma separated the rings of medullary vascular bundles and peripheral vascular bundles and was continuous in all species except *P. betle* (Fig. 1c). *P. betle* developed a deeply furrowed xylem cambial variant by parenchyma proliferation, which is not found in the other six species. The mucilage canals varied from absent (*P. sintenense* and *P. taiwanense*) to present in the center of the pith (*P. arborescens*, *P. kadsura*, *P. kawakamii*, and *P. kwashoense*; Fig. 1a, d–f) or present in the pith and inner cortex (*P. betle*; Fig. 2a). The maximum and minimum width of rays differed from each other in all species except *P. sintenense*, which had rays of a uniform width (Fig. 1g). The medullary vascular bundles persist but their growth is eventually minimal, while the peripheral vascular bundles continue to develop as the stem increases in diameter, and secondary thickening is restricted to the peripheral vascular bundles (Fig. 2a–d) due to the fascicular cambia that are located between the phloem and xylem of the peripheral vascular bundles.

By comparing different sizes of stems in *P. betle* (Fig. 2a–d) and *P. kadsura* (Fig. 2e–h), two different series of vascular bundle development were observed. In the first type of development, much of the stem’s volume is parenchyma, with one central mucilage canal is located in the pith, and eight additional mucilage canals in the inner cortex (Fig. 2a). The medullary vascular bundles experience secondary growth from a fascicular cambium until they reach a certain size, while the number of medullary vascular bundles increases (Fig. 2b), the cortical vascular bundles do not grow appreciably, and a sclerenchymatous ring thickens (Fig. 2c). Then the growth of medullary vascular bundles is essentially minimal, while the peripheral vascular bundles produce xylem and phloem from a fascicular cambium, eventually exceeding the medullary vascular bundles, and wide rays fully separate the sclerenchymatous ring, which becomes discontinuous (Fig. 2d). The species *P. betle* showed this type of development.

In the second type of development, much of the stem’s volume is also parenchyma, and only one mucilage canal is present, located in the center of the pith (Fig. 2e). Approximately ten medullary vascular bundles and a sclerenchymatous ring are in the inner cortex, and all the cortical vascular bundles apparently develop. Both medullary vascular bundles and cortical vascular bundles continue to grow (Fig. 2f), and a ring of sclerenchyma gradually forms (Fig. 2g). Then, the growth of medullary vascular bundles becomes minimal, while the peripheral vascular bundles continuously grow from a fascicular cambium. The medullary vascular bundles scarcely change while the peripheral vascular bundles soon exceed the medullary vascular bundles (Fig. 2h), wide rays fully develop, and the sclerenchymatous ring remains continuous. The species *P. kadsura* belongs to this type.

The epidermis was one of the diagnostic features for identifying these lianescent species. *P. betle* had noticeable corky ridges (periderm) (Fig. 1b, c) on larger stems, and this characteristic was not found in the other six species. All seven *Piper* species climb via adventitious roots. Based on the characteristics of the stem transverse sections, we established a key to the seven climbing species, allowing species identification when leaves are not present.

**Table:** 

1.	Sclerenchymatous ring discontinuous; ring of mucilage canals in the cortex	*P. betle*
–1.	Sclerenchymatous ring continuous; mucilage canals central only or absent	2.
2.	Mucilage canals absent	3.
–2.	Mucilage canals in the center of the pith	4.
3.	Phloem rectangular to triangular; stem transverse section oblate	*P. taiwanense*
–3.	Phloem arcuate; stem transverse section transversely elliptic	*P. sintenense*
4.	Phloem triangular	*P. kadsura*
–4.	Phloem arcuate	5.
5.	Number of medullary vascular bundles approximately 6–7	*P. kawakamii*
–5.	Number of medullary vascular bundles > 8	6.
6.	Peripheral vascular bundles approximately 30–31, secondary xylem with, diffuse-porous vessels	*P. arborescens*
–6.	Peripheral vascular bundles approximately 34–41, secondary xylem with ring-porous vessels	*P. kwashoense*

## Discussion

Cambial variants of liana stems are divided into two types: those that originate from a single cambium and those from multiple cambia (Angyalossy et al. 2012). Members of Piperaceae such as *Manekia obtusa* (Miq.) T. Arias, Callejas and Bornst produce the external vascular cylinders type (Angyalossy et al. 2015); in the present study, all seven *Piper* species also developed two series of primary vascular bundles separated by a ring of sclerenchyma. The outer irregular series of primary vascular bundles, or cortical vascular bundles, always developed near the cortex or periderm. The inner irregular series of vascular bundles, the medullary vascular bundles, were always found around the center of the stem. The stem of *P. gorgonillense* produced wide, lignified rays, with solitary or clustered vessels (Trueba et al. 2015), and the stem of *P. sarmentosum* has a discontinuous ring of collenchyma in the outer cortex (Raman et al. 2012); these characteristics were not observed in this study. The stem of *P. obtusilimbum* has a typical arrangement of tissues (Tepe et al. 2007), but lacks mucilage canals in the pith or in the inner cortex.

The stem of *P. excelsum* had also two cylinders of vascular bundles, but two medullary bundles, rather than a mucilage canal, occupied the very center of the pith (Beck 2011). The characteristic of a thick cylinder of tracheary elements that formed radially extended regions from cambium activity in *P. excelsum* (Beck 2011), *P. amalago* (Santos et al. 2015), and *P. nudibracteatum* (Trueba et al. 2015) was also seen in *P. kadsura*. The stem of *P. retrofractum* was circular in transverse section (Saraswathy et al. 2013), unlike the oblate, transversely elliptical stems of the seven species in the present study.

The stem of *P. betle* has a ring of mucilage canals located between the cortical and medullary rings of primary vascular bundles (Raman et al. 2012); in this study, eight additional mucilage canals were found in the inner cortex. The stem of *P. kadsura* just has a central mucilage canal in the pith, as described in *P. excelsum* by Beck (2011). Though we did not observe the development process of the other five species in Taiwan, the two diagnostic features, a continuous sclerenchymatous ring and only a central mucilage canal (see Table 2), were also found in the these species, indicating that their development process is similar to that of *P. kadsura*. However, the development of the other five species was not observed, and although the two diagnostic features were the same, further study is needed to confirm that the same pattern of development occurs.

We observed that the growth of medullary vascular bundles continues but gradually becomes minimal, while the peripheral vascular bundles continue to grow in these seven *Piper* species. This result is consistent with a previous report (Trueba et al. 2015) that secondary thickening is actually restricted to the peripheral vascular bundles in species undergoing secondary growth. The variations in the activity of the medullary vascular bundles and the peripheral vascular bundles result in unusual distribution patterns of xylem and phloem, which influences stem shape based on the shape of the peripheral vascular bundles (Beck 2011). The function of the flattened stems of lianas is related to their climbing habit, where they are either appressed to or leaning on the supporting tree’s trunk; this habit is entirely restricted in epiphytic lianas (Obaton 1960). The seven *Piper* species climb via adventitious roots and form a cambial variant type with flattened stems or oblate in that the broad face of the flattened axis contacts branches of trees (Carlquist 2001). The flattened stems as well as adventitious roots seem related to an epiphytic habit. More than two vascular bundle rings or two development types might be seen in stem of larger diameter, so it is valuable to observe more details in further studies.

Cambial variants in Piperaceae include successive cambia, xylem in plates, xylem dispersed by parenchyma divisions, intraxylary phloem, included phloem (interxylary phloem), phloem arcs/wedges, and secondary growth of external primary vascular bundles (Metcalfe and Chalk 1985; Caballé 1993; Carlquist 1991, 2001, 2013; Isnard et al. 2003; Acevedo-Rodríguez 2005; Jacques and de Franceschi 2007; Angyalossy et al. 2012). In woody vines, certain segments of the interfascicular cambium produce secondary parenchyma whereas a fascicular cambium produces typical proportions of xylem and phloem, as in *Aristolochia* (Aristolochiaceae) (Beck 2011). The xylem is furrowed by arcs or wedges of phloem derived from portions of the cambium that produce less xylem and more phloem (Angyalossy et al. 2015). However, furrowed xylem may develop from phloem wedges or secondary parenchyma proliferation. Carlquist (1991) defined rays as having very wide, thin-walled cells with limited subdivision during secondary growth. Wide rays develop in Piperaceae, and during secondary growth, the rays do not subdivide very much. Indeed, there may be little difference between them. Wider rays in lianas allow the stems to experience torsion without sustaining damage to vessels and contribute to liana stem flexibility (Putz and Holbrook 1991). The functional significance of wider rays in these seven *Piper* species needs to be checked.

In the present study, we found that deeply furrowed xylem in *P. betle* was produced from parenchyma proliferation (Fig. 1c). This deeply furrowed xylem cambial variant has not been reported previously. Twenty-two families develop xylem in plates (also called radiating plate xylem), including Piperaceae. Carlquist (2001) stated that in Piperaceae, combinations of cambial variants were common, such as secondary growth of external primary vascular bundles combined with xylem in plates, so two cylinders of vascular bundles combined with deeply furrow xylem and xylem in plates in *P. betle* is another special cambial variant type. In the stems we examined, the xylem was beginning to furrow in *P. kadsura*, *P. sintenense*, and *P. taiwanense*, indicated that these three species will develop deeply furrowed xylem in older stems. In summary, the seven *Piper* species in Taiwan produce cambial activity that results in large, radially extended regions of secondary vascular tissues, which are separated from each other by wide, medullary rays of primary parenchyma.

## Conclusions

The arrangement of two concentric cycles of bundles separated by a sclerenchymatous ring is typical of *Piper* in Taiwan. The genus displays a cambial variant with external primary vascular bundles combined with xylem in plates and wide medullary rays. The stems of these seven species are irregular in conformation and generally oblate, or flattened in cross section. A continuous sclerenchymatous ring is present in all species except *P. betle*, where it is discontinuous, with an additional ring of mucilage canals. The mucilage canals varied from absent, central in the pith, or present in the pith and inner cortex; mucilage canals may be diagnostic for certain species. The deeply furrowed xylem cambial variant only appears in *P. betle* and is characterized by an interfascicular cambium that forms secondary parenchyma.

## Additional file

**Additional file 1: Appendix.** Terminologies of transverse section of stems in the family Piperaceae.
