# Supplementary material for: Cambial variations of Piper (Piperaceae) in Taiwan
Source: Bot Stud. 2017 Mar 29;58:17. doi: 10.1186/s40529-017-0172-z (PMC5432890; doi:10.1186/s40529-017-0172-z)
Supplement: Supplementary file 1 — Additional file 1: Appendix. Terminologies of transverse section of stems in the family Piperaceae. [file 40529_2017_172_MOESM1_ESM.docx]

Appendix 1 Terminologies of transverse section of stems in the family Piperaceae. The explanation of terms follows Chiang (1973), Metcalfe and Chalk (1979, 1985), Carlquist (1991), Tepe et al. (2007), Beck (2010), Raman et al. (2012), Saraswathy et al. (2013), Yang and Chen (2015), Angyalossy et al. (2015), and Santos et al. (2015).

1. collenchymas, collenchymatous outer cortex: common develop in the peripheral regions of stem.

2. cortex: between the vascular system and the epidermis in stem.

3. cortical bundle, cortical whorls of vascular bundles: vascular bundles develop in the cortex in the transverse section of stem.

4. endodermis: a specialized, single layer of cells enclosing the vascular regions of some stems.

5. epidermis: the outer layer of cells of the primary body of a plant.

6. furrowed xylem: secondary xylem is lobed or long narrow groove, while the outline of the axis remains more or less circular.

7. medullary rays: the broad regions of secondary parenchyma between elongate masses of secondary tracheary tissues.

8. medullary vascular bundles, medullary whorls of vascular bundles: vascular bundles located in the pith.

9. mucilage canal: a canal containing mucilage or gum.

10. parenchymatous pith; pith parenchyma: ground tissue in the center of a stem composed of parenchyma.

11. parenchyma proliferation: ground tissue rapidly increase in numbers.

12. pericycle, pericycle fibres: the tissue region located between the primary vascular tissues and endodermis, and the cell wall is often lignified.

13. periderm: secondary protective tissue that replace the epidermis in stems; consists of phellem (cork), phellogen (cork cambium), and phelloderm.

14. peripheral vascular bundles: vascular bundles located in the pericycle.

15. phloem: the food-conducting tissue of vascular plants which is composed of sieve elements, various kinds of parenchyma, fibers, and sclereids.

16. rays: produced by the interfascicular cambium.

17. sclerenchyma, sclerenchymatous ring, sclerenchymatous cylinder: a tissue composed of sclerenchyma cells, also a collective term for sclerenchyma cells in the primary plant body; includes fibers, fiber-sclereid and sclereid.

18. secretory cell: a living cell specialized with regard to secretion of one or more, often organic, substance.

19. vascular bundle: a strand of vascular tissues, usually primary xylem and phloem.

20. vascular cambium: a lateral meristem from which secondary xylem and secondary phloem are produced in the stem. Periclinal divisions in cambial initials produce cells, some of which differentiate into phloem cells, others of which differentiate into xylem cells.

21. xylem: a complex tissue of parenchyma and tracheary elements that function in the longitudinal transport of water and minerals.

22. xylem in plates: axial vascular elements in segments, in which axial elements of xylem and phloem are divided in segments separated by large portions of xylem and phloem rays; fibrous xylem is separated by thin-walled ray cells.
